# Novel Therapies Boosting T Cell Immunity in Epstein Barr Virus-Associated Nasopharyngeal Carcinoma

**DOI:** 10.3390/ijms21124292

**Published:** 2020-06-16

**Authors:** Sarah Renaud, Anthony Lefebvre, Serge Mordon, Olivier Moralès, Nadira Delhem

**Affiliations:** 1 INSERM, CHU-Lille, U1189—ONCO-THAI—Assisted Laser Therapy and Immunotherapy for Oncology—ImmunoPDT and Immunotherapy of Cancer Department, University of Lille, F-59000 Lille, France; sarah.renaud@immune-insight.com (S.R.); anthony.lefebvre@ibl.cnrs.fr (A.L.); serge.mordon@inserm.fr (S.M.); 2 Immune insighT, Institut de Biologie de Lille, 59021 Lille, France

**Keywords:** therapy, T cell, immunity, EBV, nasopharyngeal carcinoma

## Abstract

Nasopharyngeal carcinoma (NPC) is a malignant tumour of the head and neck affecting localised regions of the world, with the highest rates described in Southeast Asia, Northern Africa, and Greenland. Its high morbidity rate is linked to both late-stage diagnosis and unresponsiveness to conventional anti-cancer treatments. Multiple aetiological factors have been described including environmental factors, genetics, and viral factors (Epstein Barr Virus, EBV), making NPC treatment that much more complex. The most common forms of NPCs are those that originate from the epithelial tissue lining the nasopharynx and are often linked to EBV infection. Indeed, they represent 75–95% of NPCs in the low-risk populations and almost 100% of NPCs in high-risk populations. Although conventional surgery has been improved with nasopharyngectomy’s being carried out using more sophisticated surgical equipment for better tumour resection, recent findings in the tumour microenvironment have led to novel treatment options including immunotherapies and photodynamic therapy, able to target the tumour and improve the immune system. This review provides an update on the disease’s aetiology and the future of NPC treatments with a focus on therapies activating T cell immunity.

## 1. Nasopharyngeal Carcinoma

Nasopharyngeal carcinoma (NPC) is a malignant tumour of the head and neck that originates from the epithelial tissue lining the nasopharynx. Every year, over 80,000 new cases of NPC are diagnosed and 50,000 NPC-related deaths are recorded worldwide [1]. NPC typically develops on the mucosa that lines the nasopharynx epithelium. The most common forms of NPCs are those that originate from the epithelial tissue lining the nasopharynx. Indeed, they represent 75–95% of NPCs in the low-risk populations and almost 100% of NPCs in high-risk populations [2].

Based on the current World Health Organisation (WHO) pathologic classification, NPCs are grouped into keratinising squamous cell carcinoma (KSCC) and non-keratinising carcinoma. The latter group is further subdivided into non-keratinising differentiated carcinoma (NKDC) and non-keratinising undifferentiated carcinoma (NKUC) [3,4]. 

Patient’s five-year survival rate remains low as it is often diagnosed at late stages (30–40%) [1,5]. Although radiosensitive, new therapies are needed to lower the mortality associated to NPC. New anti-tumour treatments include innovative surgical methods, immunotherapies, and photodynamic therapy. In this sense, the authorisations to market immunotherapies used alone or in combination have recently been approved in tumours of the ortho-rhino-laryngology sphere. The main objectives of this review are to provide update on the disease’s aetiology and the future of NPC treatments.

## 2. NPC Epidemiology 

In most of the world, NPC is a rare disease affecting fewer than 1 out of 100,000 people per year. Nevertheless, some regions or populations show a higher incidence of NPC. For instance, NPC has a rare incidence of <1/100,000 among Caucasians and in Europe. Some parts of South China, notably Guangdong and Hong Kong, have the highest levels of incidence in the world (15–50/100,000) [6]. The rest of Southeastern China has an intermediate incidence similar to Indonesia and Vietnam (3–8/100,000 people). It is noteworthy that Chinese people who immigrate to North America still show a higher NPC incidence than non-Chinese North Americans. Nonetheless, a decrease in NPC rates has been observed among successive generations of Southern Chinese populations living in low-risk areas such as the United States [7] and Australia [8]. Some North African countries also have a high incidence of NPC, notably Tunisia, Libya, Algeria, and Morocco (3–8/100,000). Additionally, NPC is significantly more common in Arctic regions such as Greenland and Alaska with an incidence of 3–8/100,000, mostly in people who have Inuit or Aleut heritage [7].

Interestingly, it has been described that men are three times more likely to develop NPC than women [8]. It was also found that, in low-risk groups, the incidence increases with age, which is the usual distribution risk for epithelial cancers. However, in the moderate-risk and high-risk populations, there is a peak of incidence, respectively, at young adulthood and at 45–54 years old followed by a decline in incidence at older age. It is thought that the early-age peak is due to early-life exposure to aetiologic factors that vary between cultures [7].

## 3. NPC Aetiology

### 3.1. Genetic Factors

Similar to all cancers, NPC is multifactorial and is caused by a number of etiologic factors. Other than its clear association to the Epstein-Barr virus (EBV), some environmental and genetic factors have been linked to higher risks of developing NPC. 

As mentioned above, although Chinese people who migrate outside of China show a lower NPC-incidence, they still have an increased risk in comparison to the average population. This key information confirms that environment and genetic factors both contribute to the emergence of NPC. Indeed, families with many members developing NPC have been studied, yet there are no clear genetic markers that fully elucidate the seemingly strong predisposition for NPC in these cases [9]. Studies of the HLA locus have led to understand it is important in NPC carcinogenesis. Some specific HLA haplotypes (A2, B17 and Bw46) show higher risks of NPC [10], whereas other haplotypes such as A31, B13, B27, B39 and B55 seemingly protect the person from NPC [11]. Moreover, now that the technology is sufficiently advanced, genome-wide searches for other susceptibility loci have revealed chromosome 3p21 [12] as well as the *D4S405* and *D4S3002* markers on chromosome 4 [13]. 

Another reported susceptibility locus for NPC is the *TERT/CLPTM1L*-encoding region. A 2016 meta-analysis study carried out on over 8000 people of Chinese descent identified that a variation in the *tert/clptm1l* locus (Chr 5p15.33) was linked to an increased NPC risk [14]. Indeed, the telomerase reverse transcriptase (TERT) is a subunit of the telomerase complex. A default in telomerase activity is associated to many cancers, including NPC [15]. EBV oncoprotein, latent membrane protein 1 (LMP1), has also been linked to abnormally long telomerases in NPC cell lines [16]. Additionally, cleft-lip and palate transmembrane protein-1-like (CLPTM1L) is known to be involved in *Ras*-dependent oncogenic transformation in lung cancer [17]. Thus, further studies are still needed to determine the exact mutation site and underlying mechanisms that contribute to NPC onset. 

Other loci already identified in carcinogenesis are studied for their potential involvement in NPC. When deleted, the glutathione S-transferase M1 (*gstm1*) is linked to increased NPC susceptibility in all high-risk populations [18]. Studies have also shown mutated genes encoding for cytokines or their promoters such as IL-1α [19], IL-16 [20], and IL-18 [21,22] can increase the risk on NPC.

Finally, it is well described that men are 2–3 times more likely to develop NPC than women. Thus, the involvement of X chromosome variations has been hypothesized. However, the study of the X-chromosome is generally left out in genome studies, as it is far more complex to study than autologous chromosomes. The X-inactivation phenomenon in females helps balance out the allele dosage between genders and silences one of the two copies of the gene [23]. This makes studying potential susceptibility loci on sexual chromosomes very difficult. Nonetheless, the difference in NPC risk observed between genders could also be explained by the culturally unequal exposure to environmental contributing factors. 

### 3.2. Environmental Factors

Dietary habits were first put forward as an etiological factor for NPC by John Ho [24]. He suggested that prolonged and repetitive consumption of Cantonese-style salted fish contributed to NPC onset. Indeed, this was later confirmed in a study on rats, as Cantonese-style salted fish enhanced nasal cavity carcinomas and NPC [25,26,27]. It was further discovered that this food contains nitrosamines and their precursors that are highly carcinogenic [28,29] and contain EBV-activating factors [30]. The same result was also found in the salted fish consumed in Greenland [30,31]. Likewise, high incidence rates in North African countries where nitrosamines/precursors and EBV-activating substances were found in salt-preserved foods such as harissa, qaddid and touklia [30,31,32,33].

The risk of developing cancer in the upper respiratory tract is commonly linked to smoking cigarettes [34]. However, studies have been inconsistent regarding the impact of smoking on NPC incidence [35,36,37,38]. As the nasopharynx traps primarily medium-size particles (5–10 µm), maybe the nasopharynx epithelium is less sensitive to cigarette smoke-induced carcinogenesis. However, studies carried out on British, North American and Chinese workers who are regularly exposed to coal combustion smoke and wood dust showed greater risks of NPC [37,39,40,41,42]. Moreover, alcohol consumption was not initially thought to contribute to NPC [35,38], but after re-evaluation it was concluded that extensive use of alcohol significantly increases NPC risks [43,44].

### 3.3. Viral Factors: Epstein-Barr Virus

NPC is widely recognised as an EBV-associated malignancy. EBV is part of the *Gammaherpesvirinae* family and is known as Human Herpes Virus-4 (HHV-4). As with all member of this family, it is a double-stranded DNA virus containing 85 genes found in the nucleoid. The nucleus-like region is bound by an icosahedral nucleocapsid that measures 100–120 nm in diameter and is made up of 162 capsomeres. The space between the nucleocapsid and the outer envelope is lined with the tegument, a protein-rich matrix. This envelope contains proteins and surface glycoprotein projections that originate from the cell’s nuclear membrane. The projections help the virus bind to the target cell.

Nonetheless, as EBV infects most of the world’s population, it was hypothesized that certain strains of EBV were responsible for specific NPC endemic regions. Indeed, some EBV variants can be significantly correlated with high- incidence of NPC in endemic regions [45,46]. Feng found that a single nucleotide polymorphism in the EBV genome (*locus 155391*: G>A) can be linked to increased NPC susceptibility in Southern China [47]. Furthermore, infection with multiple strains of EBV has also been described using heteroduplexes [48]. Evidence shows a selective presence of different EBV strains in the saliva and peripheral blood of the same patient [49]. This variation is thought to occur during the high replication phase of the virus. Different strains show varying expressions of EBV nuclear antigen-1 (EBNA1) which might affect its recognition by the host’s immune system [50]. This brings an evolutionary advantage to EBV as this diversity increases the number of targets for the host’s cell making its eradication much more strenuous.

The Epstein-Barr virus latently infects more than 90% of the world’s adult human population and its association with NPC is thought to be mediated by an interplay of environmental (dietary, smoking, co-infectious) factors and genetic predisposition (high-risk HLA allotypes). In NPC, the EBV virus expresses a type II latency program and is present in virtually all poorly differentiated and undifferentiated non-keratinising (WHO type II and III) NPCs.

EBV-associated NPC expresses a type II latency program, and tumour cells typically express the latent membrane proteins 1, 2A, and 2B (LMP1, LMP2A, and LMP2B) and EBNA1, all of which have limited immunogenicity. In addition, several EBV non-coding RNAs primarily EBER1 and EBER2, and BamHI-A rightward transcripts (BARTs) and BamHI-A rightward frame 1 (BARF1) of EBV are expressed abundantly and are detected consistently in NPC [51,52,53,54,55,56].

EBNA1 is frequently expressed in NPC and is a dominant target for CD4 T cells. LMP1 and LMP2 are expressed in approximately 50% of all NPC tumours. Although LMP1 is poorly immunogenic, LMP2 proteins are sufficiently more immunogenic hence they are now putative targets for EBV-directed immunotherapy, such as cytotoxic T cells [52,57,58,59]. NPC occurs in immunocompetent individuals, and it is likely that immunological pressure results in the expression of a limited array of EBV antigens. These proteins maintain cellular transformation in malignant cells and their poor immunogenicity is expected to play a role in promoting immune escape by EBV+ malignant cells [60,61]. EBNA1 can be detected in all EBV-associated malignancies including NPC [59].

NPC-related EBV antigens LMP1, LMP2A/B, EBNA1, EBER and EBV-encoded RNA each have distinct effects on growth, differentiation and the host’s immune response. Collectively, they likely contribute to the development of NPC by promoting cell transformation and angiogenesis, inhibition of apoptosis, induction of stem-cell-like phenotype and enhancement of cell motility. Pressure-driven selective evolution constantly fosters the emergence of new EBV variants [62,63]. These may be more oncogenic and less immunogenic than the parental strain, with, for example, a higher tropism for epithelial cells rather than B cells, suggesting that some EBV strains may contain an increased NPC risk [64].

It is important to note that NPC, while associated with EBV and the expression EBV proteins, is an entity that encompasses a broad range of other distinct molecular aberrations that may also be targets for immunotherapies [1].

## 4. NPC Classification

Before treating the patient, the stage of the cancer must be determined using the AJCC Cancer Staging Method. This classification system takes into consideration three main factors: (T) the characteristics of the main tumour mass; (N) the status of cancer spread in the lymph nodes (LNs); and (M) the status of metastasis outside the head and neck. The patient is assessed using the chart in Table 1.

## 5. NPC Microenvironment

As a lymphoepithelioma, NPC is highly linked to the host’s immune system. It has been shown that EBV favours carcinogenesis by evading the immune response [65]. Although the patient shows a strong antiviral response coupled with a high leukocyte infiltration of the tumour, this is still not enough to fight the tumour. This immune infiltrate is mainly made up of tumour-infiltrating T cells (TILs), B cells, dendritic cells, monocytes, and eosinophils. It has been found that the infiltrating immune cells are led to the tumour site by chemokine-dependant mechanisms [66,67,68]. Indeed, it is now known that an immunosuppressive tumour microenvironment gives immunological space for the tumour to grow. This local tolerance is mediated by cytokines and regulatory immune cells that are diverted from their original purpose [68,69,70]. It was also discovered that tumour-derived exosomes contribute to maintaining tumour immune tolerance by favouring regulatory T cells (Tregs) [67], among other mechanisms [71]. EBV thrives on this immunosuppressive tumour microenvironment and its key contributors are viral proteins such as EBNA1 and LMP1 [72,73].

## 6. NPC Conventional Treatments 

Due to the deep-seated localisation of NPC, surgery to remove the tumour is generally not applicable. However, nasopharyngectomy is a possible treatment for locally recurrent NPC. Different surgical methods exist including the maxillary swing, midface degloving, transpalatal, transmaxillary and trans-infratemporal fossa approaches, but all of these surgeries remains invasive [74]. In recent years, there has been much progress in minimally invasive surgical techniques. Endoscopic nasopharyngectomy was the first technique used for the resection of early stage recurrences and has shown promising results [75]. Indeed, a study including 91 patients at different stages (30 rT1, 13 rT2, 29 rT3 and 19 rT4) having undergone an endoscopic nasopharyngectomy (ENPG) showed good results with an overall survival rate at two and five years of 64.8% and 38.3%, respectively, and disease-free survival rates of 57.5% and 30.2%, respectively. At 109 months follow-up of the 91 patients, 42 were disease-free, 10 were alive with stable disease and 30 had died [75].

ENPG techniques have also been used in later recurrences including rT3 and rT4 diseases [76]. In one study, 15 patients with recurrent rT3 or rT4 NPC underwent ENPG. Overall survival at two years, disease-free survival and disease-specific survival were 66.7%, 40% and 73.3%, respectively. In addition, no post-operative complications were observed [76]. Finally, a retrospective study including a cohort of 144 patients with rT1 to rT3 tumours compared the efficiency of ENPG with that of intensity-modulated radiotherapy (IMRT). This study showed ENPG was more effective than IMRT for maximising five-year survival (77.1% vs. 55.5%), preserving quality of life and minimising complications following treatment (12.5% vs. 65.3%) [77].

Another minimally invasive approach is the use of robotics in nasopharyngectomy. Tsang et al. used this technique with the da Vinci S system (Intuitive Surgical Inc., Sunnyvale, CA, USA) on a cohort of twelve patients [78]. At the end of this treatment, overall survival at two years and disease-free survival were 83% and 61%, respectively, showing good efficiency. However, this technique often requires splitting the palate in an irradiated field, which has hindered its used. Other, more flexible robotic systems have emerged to overcome these problems. For instance, the use of the Flex^®^ system (Medrobotics, Raynham, MA, USA) in preclinical studies allowed a transoral palate-sparing approach to the nasopharynx [79]. 

On the other hand, a craniofacial resection has been proposed for the removal of advanced rT3 and rT4 recurrent tumours [80]. A monocentric study on 28 patients was performed using this technique. The overall survival at five years was 52% and 13 patients had a microscopically clear resection margin. Nonetheless, physical dysfunctions were reported in some patients including swallowing and speech impairment. Thus, these new minimally invasive surgical approaches provide novel treatment options for NPC patients. However, some can leave side effects and have a significant impact on patient’s quality of life. 

When diagnosed at early stages, NPC is classically treated with either radiotherapy and/or chemotherapy with over 90% five-year survival rate [81]. In a Phase III clinical trial (INT-0099), it was found that coupling fluorouracil (5-FU) and cisplatin chemotherapy with radiotherapy (CRT) increased overall survival by 31% [82]. However, if the tumour has locally spread, the five-year survival rate falls to 50–70% and even lower in cases of distant metastases. Although palliative chemotherapy does have an initial 80% response rate, the disease eventually stops responding to treatment after 12–18 months [81]. Unfortunately, patients that no longer benefit from CRT find themselves at a therapeutic impasse. However, efforts are currently underway to develop new treatments. 

## 7. NPC Novel Therapies 

### 7.1. Targeted Therapies

Targeted therapies were being tested for all types of cancers at the turn of the century. These molecular agents target the increased cell growth and resistance to cell death attributed to tumour cells. Monoclonal antibodies are used to target the EGFR pathway and block multiple key effector Tyrosine-kinases. In lung cancer, they are now the gold standard treatment for patients. However, clinical trials have shown only modest advantages for NPC patients. Major toxic side-effects were recorded including some cases of Grade 5 tumour haemorrhaging with pazopanib [83] and sunitinib [84] leading to the premature end of this last trial. Thus, the multi-kinase inhibitors’ lack of effectiveness explains why they are not used for the treatment of NPC at this day.

### 7.2. Immunotherapies

One of the main characteristics of NPC is the presence of a massive leukocyte infiltrate within the primary tumour. The latter consists of tumour-infiltrating T cells (TILs), B cells, dendritic cells, macrophages, monocytes, and eosinophils whose recruitment is facilitated by the production of inflammatory cytokines by tumour cells [68,85]. Cytokines such as IL-1α [85] and chemokines (MIP-1α) [68] by tumour and immune cells is particularly involved in the recruitment of immune cells at the tumour site. Moreover, it was observed that local immunosuppression was mediated by both immunosuppressive cytokines, such as IL-10, and regulatory cells. Among these regulatory cells, the presence of Tregs in TILs has been described in patients inhibiting the effector T cell response [86]. It was also discovered that EBV proteins play a role in local immunosuppression, notably LMP1, which promotes the expansion of myeloid derived suppressive cells (MDSCs) [87]. 

**Figure 1:**
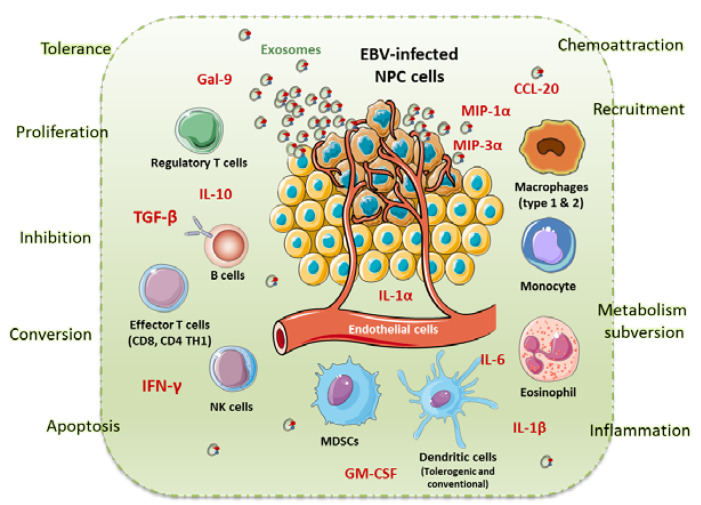
Nasopharyngeal Carcinoma immune microenvironment (inspired by Tsang et al. [88]). This figure illustrates the presence of multiple immune cells and cytokines in the tumour microenvironment (TME) of NPC. Many proinflammatory cytokines including MIP1-α, MIP3-α (CCL20), interferon (IFN)-γ, interleukin (IL)-6, GM-CSF (Granulocyte-macrophage colony-stimulating factor), IL-1-α and IL1-β are present in the TME. MIP3-α is produced by the NPC cells and is a chemo-attractant for lymphocytes and dendritic cells through CCR6. IL1-α, IL1-β, IL-6 and GM-CSF are also produced by NPC cells inducing a proinflammatory microenvironment. TGF-β and IL-10 are important immunosuppressive cytokines that promote the tumour’s immune evasion. NPC cells release massive quantity of exosomes, which express Galectin 9 and CCL20 to avoid immune detection. Finally, NPC cells increase the release of IL1-β, IL-6 and GM-CSF in the TEM to induce the expansion of myeloid derived suppressive cells (MDSC), which in turn promotes immune suppression.

Figure 1 summarises the immune microenvironment within NPC. This figure, inspired by Tsang et al. [88], shows the presence of multiple immune cells and cytokines in the tumour microenvironment (TME) of NPC. Many proinflammatory cytokines including MIP1-α, MIP3-α (CCL20), interferon (IFN)-γ, interleukin (IL)-6, GM-CSF (Granulocyte-macrophage colony-stimulating factor), IL-1-α and IL1-β are present in the TME. MIP3-α is produced by the NPC cells and is a chemo-attractant for lymphocytes and dendritic cells through CCR6. IL1-α, IL1-β, IL-6 and GM-CSF are also produced by NPC cells. TGF-β and IL-10 are important immunosuppressive cytokines that promote immune evasion for NPC cells by suppressing the proliferation and activity of tumour infiltrating leucocytes. NPC cells release massive numbers of exosomes to evade immune detection (Figure 1). LMP1, Galectin 9 and CCL20 were found in these exosomes. NPC exosomes induced the apoptosis of effector T cell. NPC-derived exosomes can also recruit Tregs through CCL20 to promote their suppressive activity and induce the conversion of conventional T cells into Tregs. Finally, LMP1-mediated metabolic reprogramming of NPC cells has been shown to increase the release of IL1-β, IL-6 and GM-CSF in the TME to induce the expansion of MDSCs, which in turn promotes immune suppression. 

The majority of these components participate in the establishment of NPC immunosuppressive microenvironment, protecting it from the host’s immune response. Given the strong immunosuppressive TME and the EBV-linked nature of NPC, when the first immunotherapies were tested in clinical trials, NPC posed as a good candidate for this novel treatment. Immunotherapy engulfs many types of approaches, e.g. EBV vaccines, adoptive immune cell therapy and immune checkpoint inhibitors [1].

### 7.3. EBV-Based Strategies

We discussed above how viral proteins expressed in NPC contribute to its progression. The strategy is to target essential viral proteins or RNAs to weaken the viral drivers of NPC [89,90]. 

It is well established that NPC expresses viral proteins, mainly EBNA1-3 and LMP1-2 that are involved in carcinogenesis. However, evidence shows that the best strategies target multiple EBV proteins simultaneously. Thus, an EBV vaccine would increase the availability of viral antigens and ultimately enhance the EBV-specific immune response. Phase I studies using highly immunogenic fusion protein expressed by a recombinant virus showed an increase in the EBV-T cell population [91], whereas loading modified LMP1-2 on autologous DCs did not lead to any change in EBV-T cell numbers [92]. 

A peptide-based vaccine strategy was proposed by our team for EBV-associated malignancies including NPC [93]. Six peptides derived from EBNA1, LMP1 and LMP2 with high affinity for major histocompatibility complex class II molecules were selected. It was shown in healthy donors that the EBV-peptides induce IFN-γ-secreting CD4+ T cells and are also recognised by CD4+ memory T cells followed by IFN-γ and IL-2 secretion. Furthermore, cytotoxic EBV-specific CD4+ T-cell lines were generated on original models expressing type II latency EBV antigens (EBV-transformed T cells and monocytes) and also on lymphoblastoid cell lines (LCLs) expressing type III latency EBV antigens. In addition, granzyme B enzyme-linked immunospot assays suggested that this cytotoxic activity could be partly linked to the granule lytic pathway [93]. Strikingly, the authors showed that neither phenotypical nor functional changes in CD4+CD25+CD127(Low)-regulatory T cells were observed in response to the EBV-peptides. This avoids any future risk of aggravating a pre-existing immunosuppressive microenvironment as reported in NPC. 

Viral RNAs are also interesting targets as they are expressed in all EBV-latencies. However, their exact functions remain elusive and their diversity makes them difficult targets. Nevertheless, RNA-targeting drugs such as Ribavirin, a nucleoside inhibitor, is already being used to treat other viral diseases such as Hepatitis C and viral haemorrhagi fever, although these are RNA viruses. To our knowledge, no major developments of EBV-RNA-based treatments are currently underway. 

### 7.4. Cell Therapy

Given the strong T-cell response mounted towards EBV, it seems logical to try to enhance such an immune response. The two main leads use autologous dendritic cells or T cell that are enhanced *in vitro* and then reinjected into the patients [93]. A Phase III trial currently underway aims to treat patients with enhanced EBV-specific cytotoxic T cell after completing a first course of chemotherapy (NCT02578641) [94]. Out of the 35 patients tested, 3 showed a complete response and 22 patients partially responded. The overall response rate was 71.4% with five patients who did not require further chemotherapy treatment. Another group used autologous T cells that were presented with EBV antigens, notably LMP2, by autologous EBV+ LCLs. The cells obtained were indeed cytotoxic CD3+ CD8+ T cells that showed specific killing of EBV-LCLs. After reinjection into the stage IV NPC patients, 6 out of the 10 patients showed a control of disease progression (two with partial response and four with stable disease). Moreover, the treatment was generally well tolerated with grade 1 and 2 toxicities observed in two patients [95]. The same group hypothesised that a lymphodepletion before reinjecting the cells would help enhance the adoptive cell therapy. Unfortunately, this was not the case as administering lymphodepleting chemotherapy beforehand did not improve clinical benefit [96]. 

Nevertheless, it is worth mentioning that, although cell therapy has shown promising results, it remains very costly and technically difficult. Thus, other, cheaper and more accessible therapies are also being developed. 

### 7.5. Immunotherapy Targeting Checkpoint Inhibitors

Given that most key immune regulatory checkpoints are expressed in NPC cells, targeting them using checkpoint inhibitors seems logical. Programmed death-1 (PD-1) is found on the surface of activated B and T cells and is an inhibitory molecule that favours immune tolerance. PD-1 interacts with members of the B7 family: PD-Ligand 1 (PD-L1) and PD-Ligand 2 (PD-L2) [97]. However, other immune regulatory checkpoint molecules are also gaining interest including cytotoxic T lymphocyte-associated protein 4 (CTLA-4) [98], which is expressed on activated T cells and blocks activation molecules (CD80-CD86) found on antigen presenting cells. Indeed, it is even suggested that the expression of PD-1, PD-L1 and CTLA-4 could be used as biomarkers for prognosis and to better stratify NPC patients [99,100,101,102]. 

Table 2 summarises all the completed and ongoing clinical trials testing immunotherapy in NPC.

### 7.6. Photodynamic Therapy 

Photodynamic therapy (PDT) is a new promising treatment for cancers. It involves the administration of a photosensitiser, followed by a local illumination of tumour tissue with light of a wavelength complementary to the absorption spectrum of the photosensitiser. The photosensitiser produces reactive oxygen species (ROS) when activated by light. ROS’ in turn induce direct cytotoxicity of tumour cells as well as indirect destruction of tumour cells through vascular damage [106]. It has been reported that PDT increases the survival rate of NPC patients. 

An initial study conducted by Lofgren et al. on recurrent or persistent forms of NPC was designed to determine the efficacy of PDT in five patients who were unresponsive to radiotherapy [107]. Four patients were treated with a hematoporphyrin derivative at 2.5 mg/kg and one with potfimer sodium. After PDT treatment, three patients were tumour-free at 51 and 60 months, another patient had persistent disease and the last one had a recurrence six month later. This study showed the efficiency of this new therapy in NPC previously treated with irradiation. However, a few side effects were reported, significant headaches and minor problems of photosensitivity following sun exposure. Shortly after, Tong et al. conducted a similar study on 12 patients treated with PDT for recurrent NPC [108]. They underwent PDT with a hematoporphyrin derivative at 5 mg/kg and were exposed to 200 J/cm^2^ light. All 12 patients responded to treatment, among who three remained disease-free after 12 months and three others achieved useful palliation. The only complication observed was skin hypersensitivity. An interesting study conducted by Li et al. was designed to evaluate the clinical response of Photofrin PDT in patients with relapsed NPC. Thirty patients were divided into two groups of either Photofrin PDT or chemotherapy. Six months after treatments, PDT showed better local response and nasal cavity obstruction remission than chemotherapy [109].

Following these promising studies, other trials were carried out involving other photosensitisers. For instance, Indrasari et al. reported the efficiency of Foscan^®^ PDT in one patient with residual NPC (T4N0M0) after failing to respond to chemotherapy and radiotherapy [110]. He was treated with 0.15 mg/kg of Foscan^®^ before illumination. At the end of the therapy, a remarkable long-term response was described with no tumour progression and no lymph node metastasis. The same team conducted a similar study on 21 patients which showed that Foscan^®^ PDT is effective in treating local failures of NPC with a depth of less than 10 mm [111]. Nyst et al. reported 22 patients treatment with Foscan^®^ PDT and once again this treatment was well tolerated by patients and showed efficacy against residual or recurrent NPC [112]. Similarly, Succo et al. studied six patients with recurrent or persistent NPC who underwent Foscan^®^ PDT [113]. All patients were treated with 0.15 mg/kg of Foscan^®^ before illumination. After the first PDT, two patients are disease-free at 38 and 71 months, one patient remains disease-free after a second PDT treatment, another one is currently living with the disease and two patients died. Temporary pain during swallowing was observed in all patients.

Moreover, the effects of other promising photosensitisers such as 5-aminolevulinic acid (ALA) and its hexyl ester (ALA-H) were assessed on NPC cells [114]. Wu found that NPC tumour cells were sensitive to both 5-ALA and ALA-H PDT. They induced apoptosis and necrosis of tumour cells as well as an inhibition of the expression of matrix metalloproteinase-2, a marker for tumour metastasis. Du et al. reported the potential of hypericin as a PDT tool in the treatment of NPC/HK1 tumour cells [106]. They observed that hypericin appears to be a potent photosensitiser as it induced vascular damage and direct tumour cells killing by necrosis. These new compounds are promising but have yet to be tested *in vivo* before they can be used as a therapy.

Thus, all these studies tend to indicate that PDT has the potential to be an effective local treatment for recurrent/persistent NPC with only side effects linked to photosensitivity.

Interestingly, it has been shown that PDT shuts down the tumour microvasculature and stimulates the host’s immune system. In contrast to surgery, radiotherapy and chemotherapy that are mostly immunosuppressive, PDT causes acute inflammation, expression of heat-shock proteins, invasion and infiltration of the tumour by leukocytes and might increase the presentation of tumour-derived antigens to T cells [115]. 

Indeed, PDT produces tumour-cell destruction in the context of an acute inflammation that acts as a “danger signal” to the innate immune system. Activation of the innate immune system increases the priming of tumour-specific T lymphocytes that have the ability to recognise and destroy distant tumour cells and, in addition, lead to the development of an immune memory that can prevent recurrence of the cancer at a later time. PDT may also be successfully combined with immunomodulating strategies that are capable of overcoming or bypassing the escape mechanisms employed by the progressing tumour to evade immune attack [116].

As is well described, anti-cancer therapy is more successful when it can also induce an immunogenic form of cancer cell death (ICD). Therefore, when developing new treatment strategies, it is extremely important to choose methods that induce ICD and thereby activate an anti-tumour immune response leading to the most effective destruction of tumour cells. A very interesting work analysed whether the widely clinically used photosensitisers, photosens (PS) and photodithazine (PD), can induce ICD when used PDT. Using dying cancer cells induced by PS-PDT or PD-PDT, the authors notably demonstrated the efficient vaccination potential of ICD *in vivo*. Thus, their results identified PS and PD as novel ICD inducers that could be effectively combined with PDT in cancer therapy [117].

Otherwise, a very recent investigation from our team has suggested that PDT, which is an effective therapy in the treatment of pancreatic ductal adenocarcinoma (PDAC), also activates the immune system and could be considered as a real adjuvant for anti-cancer vaccination. The authors developed a new photosensitiser (PS-FOL/PS2) that is associated with an addressing molecule (folic acid) targeting the folate receptor 1 (FOLR1) with a high affinity (published patent: WO2019 016397-A1, 24 January 2019). Folate specifically binds to FOLR1 which is expressed in 100% of PDACs or over-expressed in 30% of cases. Interestingly, they observed a significant increase in the proliferation of activated human PBMCs and T cells when cultured with PDAC cell-conditioned media subjected to PS-FOL/PDT [118].

Moreover, in the context of squamous cell carcinoma, a very interesting study has shown that, besides causing direct cytotoxic effects on illuminated tumour cells, PDT causes damage to the tumour vasculature and induces the release of proinflammatory molecules. Indeed, pre-clinical and clinical studies on squamous cell carcinoma have demonstrated that PDT can affect both the innate and adaptive arms of the immune system. Besides stimulating tumour-specific cytotoxic T-cells capable of destroying distant untreated tumour cells, PDT leads to the development of anti-tumour immune memory. The immunological effects of PDT make the therapy more effective also when used for treatment of bacterial infections, due to an enhanced infiltration of neutrophils into the infected regions that seems to potentiate the outcome of the treatment [119].

In the field of NPC, an *in vitro* study suggested that PDT using Zn-BC-AM photosensitiser might indirectly reduce tumour growth through the modulation of cytokine production. Indeed, by examinating the effects of EBV infection on proinflammatory cytokines secretion by NPC cells after Zn-BC-AM PDT, they showed that a light dose of 0.25–0.5 J/cm^2^ on Zn-BC-AM PDT-treated HK-1-EBV cells induce a higher level of IL-1α and IL-1β secretion than the non-treated HK-1 cells. The production of IL-1β appeared to be mediated via the IL-1β-converting enzyme (ICE)-independent pathway. In contrast, the production of angiogenic IL-8 was downregulated in both HK-1 and HK-1-EBV cells after Zn-BC-AM PDT [120].

The impact of PDT on tumour reduction through cytokines has also been confirmed by studying patients with residual or recurrent NPC who had received PDTs between 2005 and 2011. Pre- and post-test examination were used to test the hypothesis of the cytokines level difference before and after PDTs. The authors described that PDT in NPC either residual or recurrence patients after receiving radiation or in chemo-radiation therapy will yield a well-responding therapy, compared to those who did not receive PDT, and inducing systemic anti-tumour response which was marked by the increased level of immune-response cytokines [121]. 

Finally, a recent study has demonstrated in mice bearing CT26 tumours that vascular PDT with redaporfin PS, using a low light dose delivered at a high fluence rate not only destroys the primary tumour but also reduces the formation of metastasis, thus suggesting anti-tumour immunity. Indeed, this *in vivo* work characterised immune responses triggered by redaporfin-PDT and demonstrated that vascular-PDT leads to a strong neutrophilia, systemic increase of IL-6, increased percentage of CD4+ and CD8+ T cells producing IFN-γ or CD69+ and increased CD4+/CD8+ T cell ratio. At the tumour bed, T cell tumour infiltration disappeared after PDT but reappeared with a much higher incidence a day later. In addition, they showed that the therapeutic effect of redaporfin-PDT is highly dependent on neutrophils and CD8+ T cells but not on CD4+ T cells [122].

Arguably, major benefits might be achieved with immunostimulating approaches that induce appropriate tissue-based inflammation. PDT and vascular-PDT in a proinflammatory regimen achieved a successful transition from innate to adaptive anti-tumour immunity and transformed the immunosuppressive tumour microenvironment into a more favourable homing for anti-tumour immunity. This therapy may offer new opportunities to improve systemic NPC treatments.

## 8. Conclusions

When diagnosed at early stages, NPC is classically treated with radiotherapy and/or chemotherapy (CRT) with over 90% five-year survival rate [81]. Unfortunately, patients that no longer respond to CRT or have a late-stage diagnosis usually have a significantly lower overall survival rate. Hence the latest surge in new treatment methods giving promising prospects for NPC care. 

Considering the surgery option for NPC, endoscopic nasopharyngectomy was the first technique used for the resection of early stage recurrences and has shown encouraging results [75]. Indeed, several studies showed that this technique was more effective than IMRT for maximising the survival, preserving quality of life and minimising complications following treatment [77]. Another minimally invasive approach is the use of robotics in nasopharyngectomy. At the end of this treatment, overall survival at two years and disease-free survival were improved and showed good efficiency. However, this technique often requires splitting the palate in an irradiated field, which has hindered its used. Other techniques such as a craniofacial resection have been proposed for the removal of advanced recurrent tumours [80]. Nonetheless, major physical dysfunctions were reported in some patients including swallowing and speech impairment. In conclusion, all these new minimally invasive surgical approaches provide novel treatment options for NPC. However, some can have serious side effects that have a significant impact on patient’s quality of life. 

Regarding targeted therapies, clinical trials have shown only modest advantages for NPC patients with also major toxic side-effects [83] leading to the premature stop of clinical trials. Thus, targeted therapies are not currently used for the treatment of NPC.

Furthermore, when the first immunotherapies were tested in clinical trials, NPC posed as a good candidate given the strong immunosuppressive tumour microenvironment and its EBV-linked nature. Indeed, it is well established that NPC expresses viral proteins, mainly EBNA1-3 and LMP1-2, that are involved in carcinogenesis. However, evidence shows that the best strategies are the ones that simultaneously target multiple EBV proteins. Thus, an EBV vaccine would increase the availability of viral antigens and ultimately enhance the EBV-specific immune response. Viral RNAs are also interesting but elusive targets that have not yet pursued in upcoming NPC therapies. 

Moreover, given the strong T-cell response mounted towards EBV, cell therapy protocols are of great intertest. The two main leads either use autologous dendritic cells or T cell that are enhanced *in vitro* and then reinjected into the patients [93]. Like all cell therapies, there are technical and financial limits, but first studies show good efficacy with limited side effects. 

Most key immune regulatory checkpoints are expressed in NPC cells which in part explains local immune tolerance towards the tumour. Thus, testing immune checkpoint inhibitors in NPC was met with high expectations. As the complex interplay of EBV and NPC continues to be unraveled, it is likely that immunotherapeutic strategies will merge into mainstream clinical practice and offer durable remissions in patients with advanced NPC who are this day incurable.

Finally, photodynamic therapy (PDT) is a new promising treatment for several cancers, including NPC. Arguably, major benefits might be achieved with immunostimulating approaches that induce appropriate tissue-based inflammation. PDT and vascular-PDT in a proinflammatory regimen achieved a successful transition from innate to adaptive anti-tumour immunity and transformed the immunosuppressive TME into a more favourable homing for anti-tumour immunity. This therapy may offer new opportunities to improve systemic NPC treatments.

In conclusion, there has recently been substantial progress in the understanding of NPC biology. Thus, the search for compelling new diagnostic tools and treatments is gaining momentum. The repertoire of treatment options is widening, resulting in lower morbidity rates for locally recurrent NPC patients. Advances in therapeutics, namely immunotherapy and photodynamic therapy, have shown promising results. With all these exciting recent advances, we are looking forward to future studies, which will further improve our understanding of NPC and significantly improve the current management of NPC patients.

## Figures and Tables

**Table 1:** TNM classification of NPC (http://headandneckcancer.org).

		Characteristics
**T Stage**	**T1**	The tumour is within the nasopharynx, or it has grown into the oropharynx and/or nasal cavity, but no extension into the parapharyngeal space (soft tissue space behind and to the side of the pharynx).
	**T2**	The tumour extends into the parapharyngeal space.
	**T3**	The tumour has grown into the bone of the skull base and/or the sinuses.
	**T4**	The tumour has grown into the skull and/or involves the cranial nerves, hypopharynx and eye socket. Alternatively, it has extended to the infratemporal fossa or masticator space.
**N Stage**	**N0**	No evidence of cancer spread to LNs ^1^ in the neck or retropharyngeal space.
	**N1**	Presence of cancer in the LNs on one side of the neck (6 cm or less in size) and above the clavicle (supraclavicular fossa). The LNs at this stage should be found in the retropharyngeal space (6 cm or less in size, one side or both).
	**N2**	Presence of cancer in the LNs on both sides of the neck (biggest LN is 6 cm or less) and above the supraclavicular fossa.
	**N3a**	Presence of a LN with cancer bigger than 6 cm.
	**N3b**	Presence of a LN of any size that is far down the neck, just above the clavicle.
**M Stage**	**M0**	No evidence of distant spread outside the head and neck.
	**M1**	Evidence of spread outside the head and neck.

^1^ LNs, Lymph Nodes.

**Table 2:** Summary of completed or ongoing clinical trials involving immunotherapy in NPC.

Phase	Status	Treatment Tested	Patient Details	Aim of the Study	Reference
I	Completed	EBV-specific adoptive T cell immunotherapy	28 relapsed or metastatic NPC patients	To determine the safety of EBV-based adoptive transfer immunotherapy in NPC	NCT00431210 [103]
I	Active, not recruiting	EBV-specific T cells (2 antigens) that have an extra T cell receptor named DNT ± chemo lymphodepletion beforehand (Cyclophosphamide and fludarabine)	14 participants with advanced NPC	To examine efficacy of EBV-specific T cells in NPC patients and determine if lymphodepleting chemotherapy before T cell infusion increases treatment efficacy	NCT02065362
I	Recruiting	CAR-T cells (recognise EpCAM)	30 NPC and breast cancer patients	Determine if treatment is well tolerated, the dosage and the adverse effects	NCT02915445
I	Completed	Using two variants of LMP2 peptide vaccine	99 patients with a high-risk of NPC recurrence	Evaluate the immunologic effectiveness of peptide immunisation in adjuvant settings in NPC	NCT00078494
I/II	Recruiting	LMP1-CAR-T cells	20 patients with EBV associated malignant tumours (nasopharyngeal neoplasms)	Evaluate safety and efficacy of designed LMP1-CAR-T cells in the treatment of EBV associated malignant tumours.	NCT02980315
I/II	Recruiting	High-activity NKs	20 NPC patients with small metastases	Assessment of the safety of high activity NKs on NPC patients	NCT03007836
I/II	Completed	Cancer stem cell (CSC) vaccine	40 metastatic NPC patients	To demonstrate that cytotoxic T cells generated after CSC vaccination are capable of specific killing of CSCs and conferring anti-tumour immunity	NCT02115958 [104]
II	Active, not recruiting	EBV-specific adoptive T cell immunotherapy	20 relapsed or metastatic NPC patients	To determine effectiveness and safety of EBV-based adoptive transfer immunotherapy in NPC	NCT00834093 [103]
II	Recruiting	Combinations of Dendritic cells and Cytokine-induced Killer Cells (DC-CIK) treatment in solid tumours	200 patients with treatment-refractory solid tumours: Colorectal cancer Renal cell Carcinoma Nasopharyngeal carcinoma Lung cancer	Aim is to investigate the efficacy of concurrent chemotherapy with DC-CIK and CIK treatment in patients with treatment-refractory solid tumours	NCT03047525
II	Recruiting	Cisplatin and CRT ± TILs	116 patients with only locoregionally advanced high-risk NPC	The Phase I results showed that TILs following CRT resulted in sustained anti-tumour activity and anti-EBV immune responses with good tolerance	NCT02421640
II	Recruiting	(cisplatin) CRT ± nivolumab	40 NPC patients ranging from low stage II to high stage IVB	Establish how well nivolumab and chemotherapy work to treat advanced NPC	NCT03267498
II	Not yet recruiting	Pembrolizumab	63 patients with detectable levels of EBV DNA in plasma after CRT. No residual disease and/or metastases	Examine efficacy and safety of pembrolizumab on NPC patients	NCT03544099
II	Recruiting	Ipilimumab and nivolumab	35 patients with advanced NPC	Test a combination of ipilimumab and nivolumab in EBV+ NPC	NCT03097939
III	Recruiting	Chemotherapy (Gemcitabine and IV carboplatin) + autologous EBV-specific cytotoxic T cells	330 participants with advanced NPC	Assess the efficacy of CTL following first line chemotherapy in prolonging overall survival of NPC patients	NCT02578641 Phase II complete trial [94]
III	Recruiting	Camrelizumab (PD-1 Antibody) after chemoradiotherapy	400 patients with stage III-IVA non-metastatic NPC	Investigate whether adjuvant PD-1 antibody treatment could improve survival	NCT03427827
II	Recruiting	Nivolumab and ipilimumab	Patients with rare tumours including NPC	Evaluate the efficacy of a combination of nivolumab and ipilimumab on hindering tumour cell growth	NCT02834013 [105]
